# Buying Time—The Immune System Determinants of the Incubation Period to Respiratory Viruses

**DOI:** 10.3390/v2112541

**Published:** 2010-11-18

**Authors:** Tamar Hermesh, Bruno Moltedo, Carolina B. López, Thomas M. Moran

**Affiliations:** 1 Department of Microbiology and Immunology Institute, Mount Sinai School of Medicine, New York, NY 10029, USA; E-Mails: Tamar.Hermesh@mssm.edu (T.H.); Bruno.Moltedo@mssm.edu (B.M.); Lopezca@vet.upenn.edu (C.B.L.); 2 Department of Pathobiology, School of Veterinary Medicine, University of Pennsylvania, Philadelphia, PA 19104, USA

**Keywords:** incubation period, stealth phase, virus, influenza, respiratory infection, cytokines, innate immune response, type I interferons

## Abstract

Respiratory viruses cause disease in humans characterized by an abrupt onset of symptoms. Studies in humans and animal models have shown that symptoms are not immediate and appear days or even weeks after infection. Since the initial symptoms are a manifestation of virus recognition by elements of the innate immune response, early virus replication must go largely undetected. The interval between infection and the emergence of symptoms is called the incubation period and is widely used as a clinical score. While incubation periods have been described for many virus infections the underlying mechanism for this asymptomatic phase has not been comprehensively documented. Here we review studies of the interaction between human pathogenic respiratory RNA viruses and the host with a particular emphasis on the mechanisms used by viruses to inhibit immunity. We discuss the concept of the “stealth phase”, defined as the time between infection and the earliest detectable inflammatory response. We propose that the “stealth phase” phenomenon is primarily responsible for the suppression of symptoms during the incubation period and results from viral antagonism that inhibits major pathways of the innate immune system allowing an extended time of unhindered virus replication.

## 1. Introduction

The incubation period is a common feature of infection by pathogenic viruses. It is defined as the time between infection by a pathogen and the onset of symptoms. Determining the incubation periods of different pathogens assists health authorities control and track the progress of an infectious disease, thus limiting the spread of the pathogen and a possible epidemic. The length of the incubation period varies according to the infectious agent, the host immunological fitness, and previous immunological experience. In humans, it is difficult to determine the length of the incubation period since the exact time of infection is usually unknown. A thorough review of the literature by Lessler *et al.* [1] showed that the reported incubation periods for human respiratory viruses ranges from around two days for influenza and human rhinovirus (HRV) to 10 days or more for measles virus (MeV).

## 2. Termination of the Incubation Period—Onset of Symptoms is Mediated by the Immune Response

The abrupt onset of symptoms following infection with respiratory viruses marks the termination of the incubation period. Flu-like symptoms are varied and described by patients as fever and chills, malaise, myalgia, sneezing, cough, runny nose, sinus pain, congestion, headache and others [2,3]. These symptoms are associated with the secretion of type I interferons (IFNs), interleukin 6 (IL-6), interleukin 8 (IL-8), interleukin 1 (IL-1), tumor necrosis factor α (TNF-α), macrophage inflammatory protein-1β (MIP-1β), interferon-γ (IFN-γ) and other cytokines [4–6].

While some of the symptoms may be directly related to the virus’ cytopathic effect (shedding of damaged epithelium can lead to airway obstruction), most of the symptoms during influenza, MeV and HRV infections are the result of the immune response to the infection [7]. The cause of the symptoms following respiratory syncytial virus (RSV) infection is controversial and it appears that both direct virus infection and the immune response play a role [8,9].

Cytokines are usually observed prior to tissue damage generated by cytotoxic T cells or direct tissue damage caused by the virus infection. Patients treated with type I IFN, TNF-α, IL-1β, IL-1α or IL-6 for various illnesses report many flu-like symptoms without actually presenting with a respiratory virus infection [10–14]. An example of the immune system’s contribution to the flu-like symptoms is the fact that administration of TNF-α or type I IFNs can cause headaches [10,11,13].

Fever is mediated by the cytokines mentioned above, mainly IL-1, and is one of the best-understood interactions between the immune system and the nervous system. Although some aspects of the relay signals are unknown, it is largely thought that these cytokines signal the hypothalamus via the peripheral nervous system to increase the thermal set point [15–18]. Other symptoms also result from the cross talk of the immune system with the nervous system. Sneezing is mediated by the trigeminal nerve. This signal is relayed to the brain stem in response to histamines secreted by leukocytes [19,20]. Coughing is mediated by the vagus nerves below the larynx and results from an inflammatory response in the lower respiratory tract [15,21,22].

Nasal discharge (rhinorrhoea) is a combination of goblet cell secretion, gland secretion, plasma exudate, and contains dead leukocytes such as monocytes and neutrophils. The observed color change (from yellow to green) is due to the granule content of these cells [23,24].

Many other cytokines, chemokines and growth factors are present at elevated levels in the virus-infected lung and in the serum, demonstrating similar kinetics to the above-mentioned cytokines. The cellular sources of these cytokines are still not completely known but both epithelial and hematopoietic cells are involved.

## 3. Cellular Sensors for Viral Recognition

Before an anti-viral response can take place in infected cells or cells that have been exposed to viral components, viral presence must be sensed. Toll-like receptors (TLRs), Retinoic acid inducible gene I (RIG-I) like receptors (RLRs) and the inflammasome complex take part in this process.

### 3.1. The TLR System

**Figure 1.**
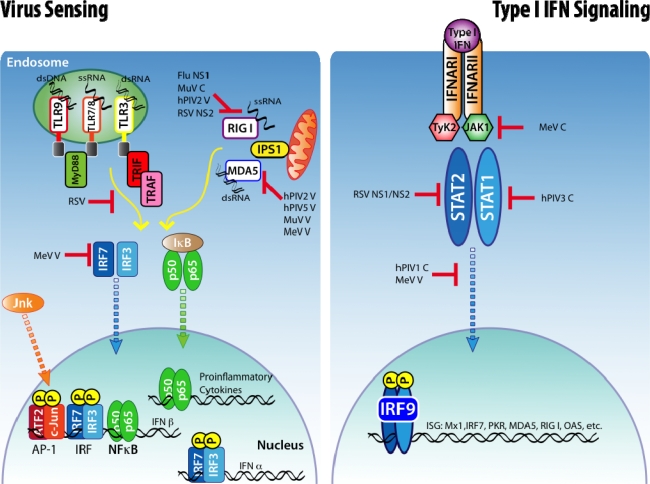
Viral antagonism to type I IFN induction and signaling. Many pathogenic viruses are able to inhibit the host cell ability to detect infection through the TLR and RLR pathways, thereby inhibiting the production of type I IFNs and other cytokines. Some viruses are also able to inhibit type I IFNs signaling.

Specialized TLRs for viral sensing are TLR-3 that recognizes dsRNA and localizes to the plasma membrane or endosome [25,26]. The endosomal TLR-7 and TLR-8 recognize viral single-stranded RNA (ssRNA) [27,28]. TLR-9 recognizes unmethylated CpG DNA of bacteria and viruses [29,30]. Some evidence suggests that TLR-4, TLR-6 and TLR-2 play a role in recognition of RSV [31,32] while MeV hemagglutinin is recognized by TLR-2 [33] (Figure 1).

### 3.2. The RLR System

The cytosolic mediators of viral sensing, the RLRs, include the RIG-I and melanoma differentiation-associated gene 5 (MDA5). RIG-I is activated by ssRNA or 5′-triphosphate double stranded RNA (dsRNA) and MDA5 by dsRNA [34–36]. Both MDA5 and RIGI signal through the mitochondrial-associated protein known as interferon beta promoter stimulator-1 (IPS-1) [36–40] (Figure 1). The role of a third member of the RLR family, the RNA helicase Lgp2, is less understood. Lgp2 has been implicated both as a negative and positive regulator of MDA5 and RIG-I function [41–43].

### 3.3. Nod-like Receptor (NLRP3) Inflammasome

The inflammasome is a protein complex composed of a number of proteins, among them caspase-1 and different Nod-like receptors (NLR)s. The main inflammasome complex involved in the response to the RNA viruses discussed in this review is NLRP3.

The inflammasome complex is required to generate the active form of the cytokines IL-1β, IL-18 and IL-33. The production of these cytokines requires two signals. Signal one is given by recognition of viral RNA as described above. This leads to increased levels of cytokines mRNA. Signal two activates the inflammasome and is sensed by NLRP3. NLRP3 is activated after exposure to ATP, dsRNA, poly I:C and various crystals such as monosodium urate [44–47].In order to produce activated cytokines, pro-IL-1β, pro-IL-18 and pro-IL-33 must be cleaved by caspase-1. Caspase-1 is part of the inflammasome complex that contains NLRP3 and the adapter apoptosis-associated speck-like protein containing a CARD (ASC) [48]. It has been shown that the NLRP3 inflammasome is required for the production of IL-1β and IL-18 during influenza infection *in vivo* [49]. It remains unclear whether the inflammasome physically senses these compounds. Recently it was suggested that influenza virus M2, an ion channel, causes changes in ionic concentration in cellular compartments which lead to NLRP3 activation [50].

## 4. Production and Signaling of Type I and III IFNs in Response to Virus Infection

The first indication of an immune response to virus infection is the secretion of type I IFNs. Type I IFNs belong to a family of cytokines consisting of one subtype of IFN-β, 13 subtypes of IFN-α and also IFN-ω, IFN-κ, IFN-ε and IFN-ν. Type III IFNs (IFN-λ) are also produced quickly after infection, and although their function and regulation is less studied than that of type I IFNs, they share similar functions. The existence of multiple IFN-α genes and the fact that virtually all viruses encode proteins that antagonize the production or response to type I IFNs emphasizes their importance during the anti-viral immune response. As we will discuss in more detail later in this review, type I IFNs secretion is delayed *in vivo* until a few days after infection and is coincident with the end of the incubation period.

### 4.1. Transcriptional Regulation of Type I IFNs

The transcriptional regulation of type I IFNs has been comprehensively reviewed [51]. In short, IFN-β is the first type I IFN to be induced following viral recognition. Transcription of IFN-β mRNA requires binding of three groups of transcription factors to the regulatory domain of the IFN promoter; NFκB, activating transcription factor 2 (ATF2)/c-Jun and interferon regulatory factors 3 and 7 (IRF-3 and IRF-7). The activation of all these factors in response to virus infection is induced by triggering either the RLR or TLR systems (Figure 1).

### 4.2. Type I IFNs Signaling

Type I IFNs signaling through its receptor leads to transcription of many interferon responsive genes (ISGs) that limit the virus replication and enhance the immune response. Secreted type I IFNs signal through the IFN-α/β receptor complex (IFNAR), composed of two transmembrane protein subunits, IFNAR1 and IFNAR2, which are present on the surface of every nucleated cell. Sensing of type I IFNs can enhance the production of type I IFNs and other inflammatory cytokines [52,53]. The dimerization of the two subunits of the IFNAR with IFN-α or IFN-β leads to activation of the intracellular kinases Jak1 and Tyk2, which phosphorylate the STAT transcription factors leading to the generation of STAT homodimers (STAT1) and heterodimers (STAT1 with STAT2). Phosphorylated STAT1 and STAT2, together with IRF-9, form a complex called interferon-stimulated gene factor 3 (ISGF3) that translocates to the nucleus and activates the transcription of ISGs [54] (Figure 1).

### 4.3. Type III IFNs

Similarly to type I IFNs, type III IFNs (IFN-λ), which in humans include IL-29, IL-28α and IL-28β, are expressed by many cell types after virus infection or TLR ligand stimulation and have similar effects to those observed with type I IFNs [55,56]. The receptor for IFN-λ (IFN-λR) is composed by one IFN-λR chain and one IL-10Rβ chain. IFN-λR also signals through the JAK-STAT pathway [57–59]. Expression of IFN-λR appears to be restricted to non-hematopoietic cells such as epithelial cells.

## 5. Inhibition of Innate Immunity by Viral Antagonists

Given that mammals have evolved a sophisticated detection and response system to viral infections, viruses have adapted to inhibit the initial recognition by the host’s immune system. Once the anti-viral response is initiated by type I IFNs signaling, it is rapidly amplified, and thus it is of great importance for the virus to delay this response as long as possible.

### 5.1. Inhibition of Interferon Induction

Viruses have evolved to inhibit IFN induction in a number of ways; the many functions of the influenza A non-structural protein 1 (NS1) have been recently reviewed [60]. Influenza NS1 inhibits RIG-I and IPS-1 signaling by forming a complex with RIG-I and ssRNA [34,61–63]. This explains the inhibition of IRF-3, NFκB, and c-Jun/ATF-2 activation observed upon infection with influenza viruses [64–66]. In addition, influenza A NS1 blocks virus detection by binding to dsRNA, thereby masking it from detection by RIG-I [67,68]. Influenza NS1 also inhibits the cellular response by interfering with the processing and export of cellular mRNA [69,70].

The paramyxoviruses’ ability to inhibit IFN has been reviewed elsewhere [71]. In brief, Sendai virus (SeV), MeV and Mumps virus (MuV) viruses V protein can block the activation of MDA5 [72–75]. Several V proteins of paramyxoviruses can inhibit IRF-3 activation [76], for example RSV NS1 and NS2 also block IRF3 activation [77]. RSV NS2 can block type I IFN induction by binding RIG-I and inhibiting downstream signaling [78].

TLR agonists are potent inducers of cytokine production. It is, therefore, surprising that very little evidence exists for inhibition of the TLR signaling pathway by the viruses discussed above. It has been suggested that certain RSV strains and MeV can inhibit type I IFN induction by TLR-7 and TLR-9 signaling. In the case of MeV, the V protein acts as a decoy substrate for the kinase IκB kinase α, competing with IRF7 [79–81]. No evidence exists for such inhibition by Influenza, HRV or human parainfluenza virus (hPIV) (Figure 1).

### 5.2. Inhibition of Type I IFN Signaling

Respiratory paramyxoviruses can inhibit the IFN signaling pathway. The C protein of hPIV1 inhibits the translocation of STAT-1 and STAT-2 to the nucleus and the activation of IRF-3 [82,83], while the C protein of hPIV3 inhibits the phosphorylation of STAT-1 [84]. Some evidence suggests the C protein of MeV acts to inhibit IFN signaling response [85]. The V protein of MeV appears to form complexes with different signaling proteins in the IFN response pathway preventing either nuclear translocation or their phosphorylation [86–91]. The NS1 and NS2 proteins of RSV can both block type I IFN and IFN-λ responses [92,93]. It is thought that STAT2 is actually degraded by NS1 and NS2 [94] (Figure 1).

Many of the proteins involved in viral recognition, type I and III IFN induction and type I IFN signaling, such as RIG-I, MDA-5, IRF7, STAT1, *etc.*, are themselves type I IFN inducible genes. By blocking IFN induction and signaling the virus also limits the enhancement of the response to infection.

## 6. Control of the Length of the Incubation Period *in vivo*

As discussed above, influenza NS1 inhibits the detection of the virus by the host thereby preventing the production of type I IFNs and other cytokines [95]. The inhibition of type I IFN production is of particular importance, since the sensing of type I IFN by neighboring cells generates an anti-viral state in these cells that limits virus propagation. Studies describing the viral proteins required for respiratory virus antagonism are limited to *in vitro* experiments, in most cases due to poor replication of antagonist deficient viruses *in vivo*. While it is difficult to extrapolate these observations to the events taking place during a natural infection, studies of influenza NS1 antagonism *in vivo* provide a model for respiratory virus inhibition of innate immunity.

### 6.1. Influenza NS1 Antagonism in vivo

A close examination of an *in vivo* influenza virus infection in mice showed that the virus replicates in the lung for almost two days without inducing an innate immune response. We defined this period between early, undetected virus infection and the first signs of an immune response as the “stealth phase”. Our group showed that the NS1 protein of influenza is responsible for the “stealth phase” by hampering cytokine production *in vivo*. Infection with a virus lacking NS1 triggers an immediate vigorous lung inflammation [96]. Two days after infection with an NS1 competent virus, a robust and abrupt immune response is initiated in the infected lungs. This event demarcates the initiation of innate immunity. The lung innate response includes the production of cytokines (e.g., IL-6, TNF-a, type I IFNs, IFN-γ and IL1-α chemokines (e.g., CCL-2, CCL-20 and KC), the recruitment of diverse cells of the immune system, and the migration of dendritic cells (DCs) to the draining lymph nodes leading to the triggering of T cell responses. This abrupt rise of chemokine is responsible for the recruitment of mononuclear phagocytes, granulocytes and other leukocytes to the site of infection. These recruited cells will play a major role in the eventual clearance of the virus.

### 6.2. Overcoming Viral Antagonism in vivo

Based on several studies, there are a number of possible mechanisms by which the immune system can be stimulated to initiate inflammation.

#### 6.2.1. Cell Death

In an inflamed tissue, the sensing of “danger signals” [97] in the form of factors released from infected necrotic or apoptotic cells may stimulate neighboring cells to produce cytokines and chemoattract other immune cells from the blood [98,99]. Viral RNA released from dying cells may stimulate TLR-7 or TLR-3 upon phagocytosis by plasmacytoid DCs (pDCs), macrophages and other cells culminating in type I IFNs production [100]. The TLR system avoids viral antagonism by rapidly sensing the virus inside endosomal compartments in uninfected phagocytes that culminates in type I IFNs and cytokine production [101,102]

#### 6.2.2. Errors in Virus Replication

Intracellular purine metabolites are released from damaged cells and include uric acid and ATP, which can stimulate the inflammasome complex to cleave pro-IL-1β and intensify the innate response [47,103–109]. The inflammasome can also activate type I IFNs production [45] and type I IFNs itself can upregulate AIM2, a protein that contains a pyrin motif that is necessary for promoting IL-1β production. IFN-γ is also involved in this signaling cascade since it can upregulate components of the inflammasome complex [110,111].

The natural process by which viruses replicate may contribute to the culmination of the stealth phase. The viral polymerase of many of the viruses discussed here is error-prone. From an evolution or natural selection standpoint, this property has the advantage of promoting rapid mutations in the viral genome, avoiding recognition by the adaptive immune response. However, it is also possible that such a process promotes mistakes in viral replication, such as the generation of mutated, less efficient viral antagonists and defective interfering virus particles (DIs) that may lead to immune recognition. It has been shown in mice that stocks of SeV with high DI content enhance the immune response [112] and MeV vaccine strains induction of type I IFNs correlates with high DI content [113].

#### 6.2.3. Priming by Type I IFNs

*In vitro* studies show that cells primed with type I IFNs are able to mount an innate response to an infecting virus, despite viral antagonism. Type I IFN signal transduction turns on transcriptional programs within cells that can decrease the inhibitory effects of the viral antagonists upon infection. Not only does the virus replicate poorly in cells primed with type I IFNs, but also primed cells can secrete pro-inflammatory cytokines more efficiently. It is known that pre-exposure of DCs to type I IFNs upregulates costimulatory molecules and major histocompatibility class I and II (MHCI and MHCII) molecules, improving their function as antigen presenting cells [114–116].

*In vivo*, lung secreted cytokines and chemokines also promote systemic awareness to the virus infection. Type I IFNs can signal to developing leukocytes and memory T cells in primary and secondary lymphoid organs such as the bone marrow and spleen to acquire an anti-viral state and enhance their function [117,118]. Such an anti-viral state is thought to functionally improve cells of the immune system before they infiltrate the lungs. Type III IFNs are also induced after respiratory virus infection and likely limit virus spread in epithelial cells [119]. Therefore, type III IFN might be induced at the end the of the stealth phase complementing the function of type I IFN. The speed at which this process occurs is controlled by the ability of the virus to suppress inflammation. This observation points out that immune modulation by the pathogen not only targets local lung immunity but also the external intervention of pre-programmed leukocytes with advantageous antiviral machinery.

Finally, the multifaceted inflammatory response can also affect non-hematopoietic cells such as uninfected epithelial cells, protecting them from infection and allowing a more vigorous response upon stimulation.

## 7. Viral Antagonism Delays the Initiation of Adaptive Immune Response

In close contact to the epithelial border is a tight network of lung DCs [120] that sense viruses and migrate along a CCR7-mediated chemokine gradient [121] to the lung draining mediastinal lymph nodes (MLNs). In the MLNs, the DCs trigger the proliferation and differentiation of virus-specific T cells [96,122–124]. Activated virus-specific effector T cells will eventually circulate back to the bloodstream and are then recruited to the respiratory tract to terminate the infection and clear the virus [125,126].

Studies tracking DC migration from the lung to the MLNs during influenza infection using fluorescent reagents that induce unspecific inflammation have shown that DCs migrate from the lung to the MLNs rapidly [127–129]. It is likely that the viral antagonist is unable to inhibit the inflammation triggered by these inflammation inducing fluorescent reagents. However, when no inflammatory agent is present in the tracking reagent, the kinetics of DC migration from the lung to the MLNs during influenza virus infection is quite slow and correlates with the termination of the “stealth phase”. DC migration begins around two days after infection when small numbers of DCs carrying viral antigens are first seen in the MLNs and reach a plateau around 3–4 days after infection [96,122,124,130]. Therefore, inhibiting inflammation for two days not only affects innate immunity but also delays the initiation of adaptive immunity.

## 8. Conclusions

**Figure 2.**
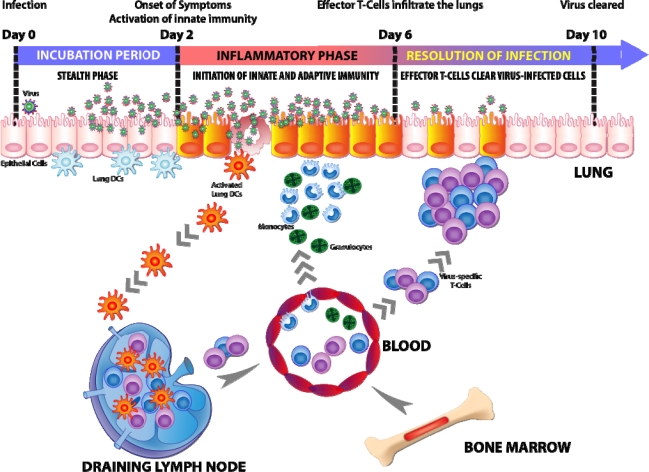
Relationship between the incubation period of influenza virus and the immune response. For the first two days after influenza virus infection, the immune response is inactive (“stealth phase”) due to viral antagonism and no symptoms are observed. The incubation period ends as symptoms abruptly appear about two days after infection when the innate immune response becomes active. The secretion of pro-inflammatory cytokines and chemokines is followed by a robust infiltration of leukocytes to the site of infection and DCs migration from the respiratory tract to the lung draining lymph nodes. The migrating DCs then present viral antigens and activate influenza specific T cells. About six days after infection, virus specific effector T cells infiltrate the lung to resolve the infection.

The incubation period is a helpful definition that describes the time between virus infection and the onset of symptoms. Based on new findings, we propose a model that describes a mechanism of the delayed symptoms (innate immune response) that is likely common to almost all known respiratory virus infections (Figure 2). The delayed rise of the innate immune response to a respiratory virus is explained by the suppression of immunity by the viral antagonist *in vivo*. The “stealth phase” is terminated by an initiating event or breakthrough that triggers type I IFN and other cytokines that serve to stimulate cells before they are infected. Type I IFN primed cells are protected from viral antagonism allowing the innate immune response to proceed. Much work must still be done to determine the factors, the sequence of events, and cell types that are relevant to accomplish the end of the incubation period.
